# Supplementary material for: Knockout of DDM1 in Physcomitrium patens disrupts DNA methylation with a minute effect on transposon regulation and development
Source: PLoS One. 2023 Mar 8;18(3):e0279688. doi: 10.1371/journal.pone.0279688 (PMC9994747; doi:10.1371/journal.pone.0279688)
Supplement: S1 Fig — A. Number of downregulated TEs in O. sativa, S. lycopersicum, A. thaliana and P. patens ddm1 mutants, as well as in P. patens dnmt mutants. B. Total number of RNA-seq reads mapped to indicated TE family types. Reads per million (RPM) values represent the average of replicates per genotype, error bars represent standard error. (DOCX) [file pone.0279688.s001.docx]

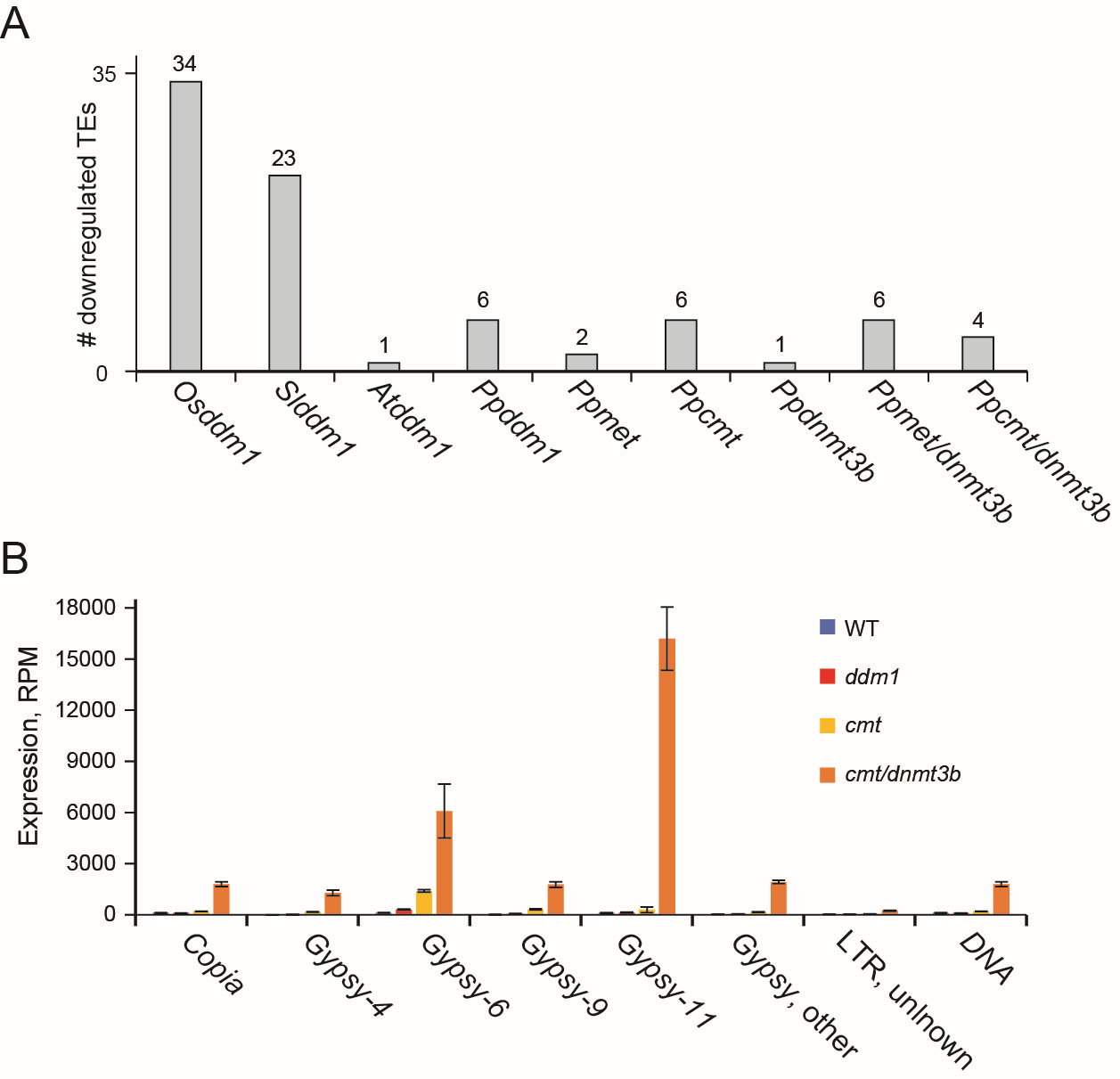


**S1 Figure. TE expression in *Atddm1*, *Slddm1*, *Osddm1*, *Ppddm1* and *Ppdnmt* mutants.**

A. Number of downregulated TEs in *O. sativa*, *S. lycopersicum*, *A. thaliana* and *P. patens* *ddm1* mutants, as well as in *P. patens* *dnmt* mutants. B. Total number of RNA-seq reads mapped to indicated TE family types. Reads per million (RPM) values represent the average of replicates per genotype, error bars represent standard error.
