# Supplementary material for: Knockout of DDM1 in Physcomitrium patens disrupts DNA methylation with a minute effect on transposon regulation and development
Source: PLoS One. 2023 Mar 8;18(3):e0279688. doi: 10.1371/journal.pone.0279688 (PMC9994747; doi:10.1371/journal.pone.0279688)
Supplement: S1 Table — Gene ID and accessions numbers for sequences used in evolutionary tree (Fig 1). (DOCX) [file pone.0279688.s002.docx]

| Gene Name | Gene ID/Accession model |
| --- | --- |
| CHR37 | AT1G05120.2 |
| CHR20/ATRX | AT1G08600.2 |
| CHR30/FRG5 | AT1G11100.4 |
| CHR18 | AT1G48310.2 |
| CHR5 | AT2G13370.1 |
| CHR3/SYD | AT2G28290.5 |
| CHR10/ASG3 | AT2G44980.2 |
| CHR2/BRM | AT2G46020.2 |
| CHR11 | AT3G06400.3 |
| CHR26/FRG3 | AT3G16600.1 |
| CHR25/RAD54 | AT3G19210.1 |
| CHR39 | AT3G54460.1 |
| CHR14 | AT5G07810.1 |
| CHR17 | AT5G18620.2 |
| CHR4/PKR1 | AT5G44800.1 |
| CHR1/AtDDM1 | AT5G66750.1 |
| PpDDM1 | Pp3c17_1952/XP_024400851.1 |
| SlDDM1a | Solyc02g062780.3 |
| SlDDM1b | Solyc02g085390.3 |
| ZmDDM1a | Zm00001d033827 |
| ZmDDM1b | Zm00001d007978 |
| OsDDM1a | LOC_Os09g27060.1 |
| OsDDM1b | LOC_Os03g51230.2 |
| MmLSH | Q60848-1 |
| HsLSH | Q9NRZ9-1 |

**S1 Table. Gene ID and accessions numbers.**

Gene ID and accessions numbers for sequences used in evolutionary tree (Figure 1).
