# Supplementary material for: Knockout of DDM1 in Physcomitrium patens disrupts DNA methylation with a minute effect on transposon regulation and development
Source: PLoS One. 2023 Mar 8;18(3):e0279688. doi: 10.1371/journal.pone.0279688 (PMC9994747; doi:10.1371/journal.pone.0279688)
Supplement: S2 Table — (DOCX) [file pone.0279688.s003.docx]

| Genotype | Mapping rate | Library size | median coverage | non-conversion rate | Tissue |
| --- | --- | --- | --- | --- | --- |
| WT | 0.68 | 40033487 | 5 | 0.0048 | Protonema |
| *Ppddm1* | 0.61 | 36504831 | 4 | 0.0045 | Protonema |

**S2 Table. Summary of BS-seq data generated in this study.**
