# Supplementary material for: Knockout of DDM1 in Physcomitrium patens disrupts DNA methylation with a minute effect on transposon regulation and development
Source: PLoS One. 2023 Mar 8;18(3):e0279688. doi: 10.1371/journal.pone.0279688 (PMC9994747; doi:10.1371/journal.pone.0279688)
Supplement: S3 Table — (DOCX) [file pone.0279688.s004.docx]

| Genotype | replicate | Mapping rate | Library size | Tissue |
| --- | --- | --- | --- | --- |
| WT | 1 | 86 | 6,047,978 | Protonema |
| WT | 2 | 86 | 7,081,383 | Protonema |
| WT | 3 | 86 | 6,702,242 | Protonema |
| *Ppddm1* | 1 | 86 | 6,703,231 | Protonema |
| *Ppddm1* | 2 | 86 | 6,679,470 | Protonema |
| *Ppddm1* | 3 | 86 | 7,558,829 | Protonema |

**S3 Table. Summary of RNA-seq data generated in this study.**
