# Supplementary material for: Knockout of DDM1 in Physcomitrium patens disrupts DNA methylation with a minute effect on transposon regulation and development
Source: PLoS One. 2023 Mar 8;18(3):e0279688. doi: 10.1371/journal.pone.0279688 (PMC9994747; doi:10.1371/journal.pone.0279688)
Supplement: Attachment — Submitted filename: Response to Reviewers Minor Revision.docx [file pone.0279688.s007.docx]

**Response to Reviewers**

Reviewer #1: In the revised version of the manuscript by Griess et al., entitled “Knockout of DDM1 in Physcomitrium patens disrupts DNA methylation with a minute effect on transposon regulation and development”, the authors have addressed most of the points raised during the initial review. The different representation of the methylation changes makes it now more logic. Several other points were answered in the response of the authors. My suggestion to make the Discussion more interesting was not followed but that is a matter of taste. I just do not understand why the data for the other Pp mutants were not added to Fig. 3E. The authors write that the total hypomethylation in Ppmet is even lower than Ppcmt or Ppddm1, but this is no argument not to include them. It would help to gain a more complete picture of the effect of all mutants.

We added methylation mutants (including Ppmet) to Figure 3E.

There are a few minor corrections necessary:
Line 211: replace “imply for” with “implies”

Replaced.

Figure 2 E and F: replace 2x “chromsome” with “chromosome”

Replaced.

Figure 2 F: change ddm1 to italic

Changed.

Reviewer #2: All my concerns have been adequately addressed except that I don't see the source or analysis methods for the H3 and H2K9me2 data in Figure 2J.

The reference for Histone H3 and H3K9me2 was added to figure legends.
